# Eating disorder psychopathology and negative affect in Iranian college students: a network analysis

**DOI:** 10.1186/s40337-022-00683-x

**Published:** 2022-11-14

**Authors:** Reza N. Sahlan, Margaret Sala

**Affiliations:** 1 Department of Clinical Psychology, Iran University of Medical Sciences, Tehran, Iran; 2 Ferkauf Graduate School of Psychology, Yeshiva University, Bronx, NY USA

**Keywords:** Eating disorder, Negative affect, Network analysis, College students, Iran

## Abstract

**Background:**

ED psychopathology is becoming more prevalent in Iran. Negative affect has been found to be an important risk factor in eating disorder (ED) onset in research conducted in Western countries, and is also emerging as a potential vulnerability factor to ED psychopathology in Iran. Network theory offers a novel framework to understand the association between negative affect and ED psychopathology in Iran. The primary aim of the current study was to use network analysis to identify bridge symptoms (i.e., symptoms that activate or weaken symptoms in another cluster) across a negative affect and ED psychopathology network among Iranian college students. We also aimed to identify core symptoms (i.e., nodes that demonstrate the strongest connections to other nodes).

**Method:**

Participants were Iranian college students (*n* = 637; 60.3% women) who completed the Farsi-eating disorder examination-questionnaire and Farsi-negative affect. We estimated a network of ED symptoms and negative affective states and identified bridge and central symptoms.

**Results:**

Hostility and shame emerged as central bridge symptoms across the negative affect and ED psychopathology clusters. The most central nodes were strong desire to lose weight, definite fear of losing control over eating, and binge eating episodes.

**Conclusion:**

The negative affective states of hostility and shame may increase vulnerability to ED psychopathology among Iranian college students. Findings have important implications for ED prevention programs that should be examined in future research.

## Plain English summary

ED psychopathology is common among Iranian college students, and negative affect may be a potential vulnerability factor to ED psychopathology in this population. However, it is unclear how specifically negative affective states may relate to ED psychopathology among Iranian college students. Therefore, we examined a network of negative affect and ED symptoms among Iranian college students (*n* = 637; 60.3% women). We found that the negative affective states of hostility and shame may link negative affect and ED psychopathology among Iranian college students. Findings have important implications for understanding the link between negative and ED psychopathology in Iran, and have potential implications for ED prevention programs in Iran that should be examined in further research.

## Background

Eating disorder (ED) psychopathology is becoming more prevalent in non-Western societies, such as Iran [1–4]. For example, Iranian college samples have been shown to adopt Western ED-related body comparisons and thin-ideal internalization [5–7] and engage in ED behaviors with similar frequency to college women in the United States (US) [7]. Therefore, it is important to examine factors that may initiate ED psychopathology in Iran. One of the most prominent risk factors and contributors to the development of ED psychopathology is negative affect [8, 9]. Specifically, negative affect has shown to play an important role in initiating and maintaining ED psychopathology [10–14]. In Iran, negative affect as well as related variables (e.g., poor emotion regulation) are also emerging as important potential vulnerability factors for ED psychopathology [15, 16].

Most research on links between ED psychopathology and negative affect, in both Iran and the West, has been conceptualized from the perspective that psychopathology symptoms result from a latent common variable (e.g., negative affect) [17]. Because of this, little is known about which individual negative affective states are most relevant to specific aspects of ED psychopathology. Whereas some studies have examined specific negative affective states (e.g., guilt, shame, fear, hostility, sadness) that may be relevant to ED psychopathology in both clinical and non-clinical samples [18–29], these studies have examined each affective states separately and have not incorporated negative affective and ED psychopathology in one integrated model.

Network theory is an alternative to traditional approaches that can increase understanding of which specific aspects of negative affect (e.g., shame vs. hostility) are most relevant to specific ED symptoms (e.g., fear of weight gain, binge eating, restraint, etc.). Network theory conceptualizes psychopathology such as EDs as interacting symptoms that activate and maintain one another [30, 31]. Network analysis can be used to elucidate connections between various vulnerability factors (e.g., negative affect) and ED psychopathology. Specifically, in network models, symptoms from one cluster (e.g., negative affect) may activate or weaken symptoms in another cluster (e.g., ED psychopathology) and vice versa via ‘bridge’ symptoms within the network. Bridge symptoms can elucidate which specific individual negative affective states (e.g., guilt vs. shame) may be linked with specific ED symptoms (e.g., binge eating vs. restricting). Network theory suggests that disrupting bridge pathways may weaken the relation between negative affect and ED psychopathology [32]. There are currently several network studies that have examined bridge symptoms between various psychological constructs (e.g., anxiety, depression, fear) and ED symptoms [33–40]. Network theory also enables for the identification of core symptoms, which are symptoms with the highest centrality that are posited to have the most overall relationships and therefore impact most other symptoms in the network. Core symptoms are suggested to be potential intervention targets (e.g., in prevention programs) because disruption of a core symptoms would weaken the overall network. Several studies [33, 39, 41–49] and a systematic review [50] examining core symptoms in EDs have been conducted, and concluded that overvaluation of weight or shape and desire to lose weight are core symptoms of EDs.

Although researchers have evaluated networks of emotional distress (e.g., depression, anxiety, fear, shame; [33–40] and EDs, to the best of our knowledge, there is only one study to date examining a network of negative affect and ED symptoms. Wong and colleagues [51] conducted a network analysis of negative affect, positive affect, and ED symptoms in a sample of US patients with EDs, and found that guilt about eating and shame were central bridge symptoms and that guilt about eating was the most central symptom [51]. However, no study to date has examined a negative affect and ED symptom network in a non-Western (e.g., Iranian) sample, and it is unclear if findings would translate in this population. There are various cultural differences in Iran that may result in differences in bridge symptoms between negative affect and ED symptoms in Iran vs. the West. For example, Iranian culture is unique because, since the 1979 Iranian revolution, modest dress (i.e., wearing Islamic-head and body coverings such as a *hijab* and a *manteau*) has been legally mandated for women and mainstream Western media. On the other hand, women in Iran do not use hijab in private places (i.e., home). This exposure may increase body dissatisfaction and/or negative affect (e.g., shame, nervousness) when seeing one’s body at home. For example, a recent study indicated that body-related shame was associated with ED psychopathology in Iran [52]. Of note, bridge symptoms between other psychological constructs (e.g., depression, anxiety) and ED symptoms have differed among individuals in Iran [4, 53] compared to those from other countries (e.g.. US; Australia; [38, 54]. Finally, the sample used by Wong and colleagues [51] mostly consisted of women, and it is unclear if findings would translate to a sample of both men and women.

Furthermore, only three studies to date have examined ED networks in Iranian individuals [4, 53, 55]. Sahlan and colleagues [4] conducted a network analysis of depression, self-esteem, and EDs in Iranian adolescents and college students, and found that desire to lose weight and discomfort when seeing one’s own body were the most central symptoms of EDs. Sahlan and colleagues [53] conducted a network analysis of social anxiety and EDs in Iranian preadolescents, and found that discomfort eating sweets was the most central symptom. Sahlan and Sala [55] conducted a network analysis of resilience and EDs in Iranian college students, and found that discomfort in seeing one’s own body, feeling guilty about eating due to shape/weight, and thinking about shape and weight making it difficult to concentrate were the most central symptoms. Of note, at least some core symptoms have differed from US, Italian and Australian samples [38, 39, 41–45, 47, 56]. For example, whereas overvaluation of weight or shape has consistently emerged as a core symptom in research conducted in the US [33, 41, 43, 46–49], it did not in one study conducted in Iran [4]. In contrast, a desire to lose weight has emerged as one of the most central symptoms in several cultures, including the US [33, 41, 43, 46–49], Italy [47], Australia [38], and in one study in Iran [4].

### Current study

The current study used network analysis to test links between specific negative affective states and ED psychopathology among Iranian college students. Our primary aim was to identify bridge symptoms within a negative affect and ED symptom network**.** We also aimed to identify central (i.e., most densely connected) symptoms as a secondary aim. Based on previous studies in US [51, 57] and Iran [4], we hypothesized that shame would bridge negative affect and ED symptoms, and that guilt about eating and desire to lose weight would be the most central symptoms in the network.

## Method

### Participants

Data from this study (*n* = 637; 60.3% women) were also used in Sahlan and colleagues’ study [4]. However, the specific aims and primary analyses of the current project are unique and have not been previously published. Specifically, bridge symptoms between negative affective states and ED symptoms have not been investigated previously. College students were recruited from two cities with diverse geographic zones and ethnicities (i.e., Tabriz [North-Western, Turk], Shiraz [South-Central, Persian]). Participants’ age ranged from 18 to 54 years (*M* = 21.89, *SD* = 3.62) and self-reported BMI ranged from 15.57 to 39.18 kg/m^2^ (*M* = 22.19, *SD* = 3.50). In the current study, 19.40% of participants reported clinical levels of ED (i.e., ≥ 2.5; Eating Disorder Examination-Questionnaire [EDE-Q], a global score; [58]. Additionally, between 1.80 and 26.70% of participants reported recurrent (i.e., ≥ 4 times during the past 28 days) binge eating, or purging (i.e., self-induced vomiting, laxative misuse, and over-exercise) (See Table 1 for more information).

**Table 1:** Means and standard deviations for eating disorder psychopathology and negative affect among college students (N = 637)

	*n*	%	–
*EDE-Q clinical threshold*^*a*^			
Yes	215	19.40	–
No	422	80.60	–
*Recurrent eating disorder behavior*^*b*^			
Binging			
Yes	168	26.70	–
No	469	73.30	–
*Self-induced vomiting*			
Yes	11	1.80	–
No	626	98.20	–
*Laxative misuse*			
Yes	13	2.30	–
No	624	97.70	–
*Over-exercise*			
Yes	74	11.80	–
No	563	88.20	–

	M	SD	Range
Age	21.89	3.62	18–54
BMI	22.19	3.50	15.57–39.18

Node label	Symptom			
*Eating disorder symptoms*				
Restrict	Restraint	1.42	2.06	0–6
Fast	Fasting	.82	1.53	0–6
Excludfood	Excluding food	.93	1.67	0–6
Foodrules	Food rules	1.31	1.85	0–6
Emptystomach	A desire to have an empty stomach	.79	1.57	0–6
Flatstomach	A desire to have a flat stomach	3.23	2.53	0–6
Foodconc	Difficulty concentrating because of thoughts of food	.89	1.52	0–6
Wtshconc	Difficulty concentrating because of thoughts of weight/shape	1.02	1.66	0–6
Fearlosecontrol	Fear of losing control over eating	1.15	1.85	0–6
Feargain	Fear of weight gain	1.75	2.23	0–6
Feelfat	Feeling fat	1.70	2.20	0–6
Desirelose	Desire to lose weight	1.91	2.40	0–6
Eatsecret	Eating in secret	.34	.85	0–6
Guilty	Feeling guilty after eating	.93	1.53	0–6
Otherseeeat	Concerns about others seeing one eat	.54	1.20	0–6
Weightjudge	Overvaluation of weight	1.84	1.97	0–6
Shapejudge	Overvaluation of shape	2.28	2.07	0–6
Upweigonself	Upset with weighing oneself more than once a week	1.12	1.62	0–6
Weightdiss	Weight dissatisfaction	2.16	2.02	0–6
Shapediss	Shape dissatisfaction	2.20	1.95	0–6
Seeself	Discomfort when seeing one’s own body	1.75	1.84	0–6
Otherseebody	Discomfort when others see one’s body	1.38	1.75	0–6
Overeat	Overeating	3.11	5.21	0–28
Losscontrol	Loss of control over eating	2.56	4.67	0–28
Binge	Binge eating	2.88	4.79	0–28
Vomit	Self-induced vomiting	.16	.79	0–10
Laxatives	Laxative misuse	.34	1.95	0–28
Compex	Over-exercise	1.45	4.33	0–28
*Negative affective states*				
Distressed	Distressed	2.73	1.25	1–5
Upset	Upset	2.93	1.26	1–5
Guilty	Guilty	2.27	1.23	1–5
Scared	Scared	2.0	1.14	1–5
Hostile	Hostile	1.77	1.05	1–5
Irritable	Irritable	2.78	1.37	1–5
Ashamed	Ashamed	2.0	1.12	1–5
Nervous	Nervous	2.68	1.34	1–5
Jittery	Jittery	2.74	1.31	1–5
Afraid	Afraid	2.05	1.18	1–5

*EDE-Q*, Eating disorder examination questionnaire

^a^The clinical threshold of the EDE-Q is a global score ≥ 2.5. ^b^Recurrent eating disorder behavior was defined as engaging in binging, self-induced vomiting, laxative misuse, and over exercise ≥ 4 times during the past 28 days.

### Procedure

All the potential participants from a broad range of departments (e.g., Psychology, Sociology, Agriculture, Tourism Management) were approached on campus or during class and were invited to participate in a study to test psychological issues among college students. Interested students completed paper–pencil questionnaires. All questionnaires were anonymous with no identifying information to protect the confidentiality of college participants. The study was approved by the Ethical Board of Iran University of Medical Sciences and all participants provided informed consent.

### Measures

#### Demographic information

Participants completed questions regarding age, gender, as well as height and weight to calculate body mass index (BMI).

#### Eating disorder symptoms

We used a Farsi version of the EDE-Q (F-EDE-Q; [1, 59]) that assesses ED symptoms over the past 28 days. The validity and reliability of the F-EDE-Q has been supported in Iran [1]. Twenty-two items are rated on a seven-point scale ranging from 0 (*No days*) to 6 (*Every day*). Five items assess the frequency of ED behaviors. Higher scores indicate a greater level of pathology. Cronbach’s α was 0.92 in this sample.

#### Negative affective states

We used a Farsi version of the 10-item Negative Affect scale [60] that assesses negative affect. The validity and reliability of the F-PANAS has been supported in Iran [60]. Responses are rated on a 5-point Likert scale from 1 (*Very slightly or not at all*) to 5 (*Extremely*). Higher scores indicate higher trait level negative affective states. Cronbach’s α was 0.86 in this sample. Of note, we also assessed positive affect, but did not include it in the model due to low stability when including positive affect.

### Data analyses

#### Missing data

One participant (0.4%) did not provide weight and height to calculate BMI. There were no missing data on any of the scales administered.

#### Item selection

We used the *goldbricker* function in the R package *networktools* to select the final items to include in the network [61]. Goldbricker compares every possible combination of correlations in the network and suggests nodes that may be redundant (i.e., measuring the same construct). Using *goldbricker* avoids inflating centrality by eliminating items that may measure the same construct [62]. We then used *best_goldbricker* to suggest which of the redundant nodes to remove. After applying goldbricker, six eating disorder items (i.e., food avoidance, preoccupation with eating, fear of weight gain, feelings of fatness, importance of shape, dissatisfaction with shape) and three negative affect items (i.e., distressed, guilty, scared) were removed. The final network had 29 nodes (22 eating disorder nodes, 7 negative affect nodes).

#### Glasso networks

We used the GLASSO estimator in the *bootnet* package in R to estimate networks. GLASSO networks estimate edges that are likely to be spurious as zero, thus resulting in a more accurate network [63]. Of note, correlations among nodes in GLASSO networks represent partial correlations in the network. We used Spearman correlations because the initial network estimated using polychoric correlations was densely connected [63]. Furthermore, Spearman correlations produce more stable networks [63].

#### Network stability

We used the *bootnet* package to estimate network stability [64]. We computed the following stability parameters: (1) an EI stability correlation coefficient (EI-coefficient); (2) an edge stability correlation coefficient (ES-coefficient), and (3) bridge EI stability correlation coefficient (BEI-coefficient). Coefficients between 0.20 and 0.50 are considered acceptable, coefficients above 0.50 and below 0.70 are considered good, and coefficients above 0.70 are considered excellent [65].

#### Centrality indices

We used the *centralityPlot* and *centralityTable* functions in the *qgraph* package in R to estimate central nodes. We calculated strength of expected influence (EI, the sum of connections between one node and all other nodes, accounting for both positive and negative connections) to identify central nodes [63]. In order to determine whether nodes with higher centrality statistics were significantly different from nodes with lower centrality statistics, we performed node centrality difference tests [65].

#### Bridge symptoms

We used the *bridge* function of the *networktools* package in R to identify bridge nodes [61]. We calculated bridge EI (i.e., the sum of the connections between one node and all other nodes, accounting for both positive and negative connections) to identify bridge nodes [66]. We used the *bootnet* package in R [65] to perform bridge EI difference tests in order to determine whether nodes with higher EI statistics were significantly different from nodes with lower values.

## Results

### Expected influence

**Fig. 1:**
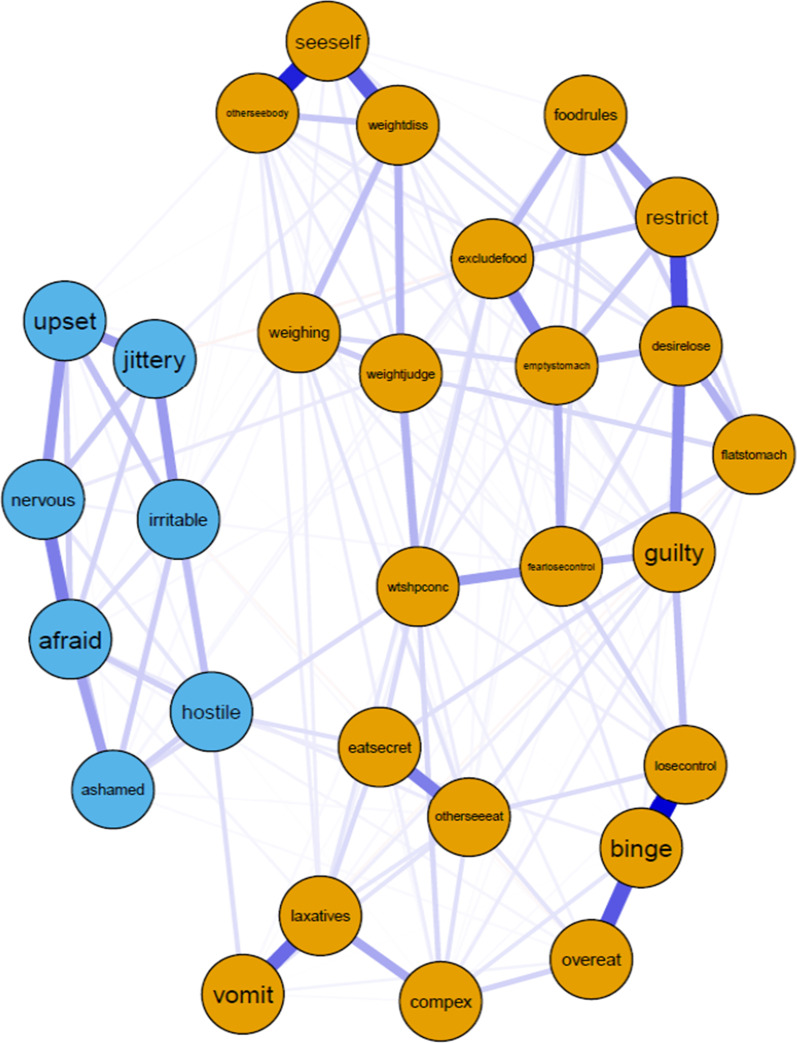
Negative affect and eating disorder (ED) symptom network

**Fig. 2:**
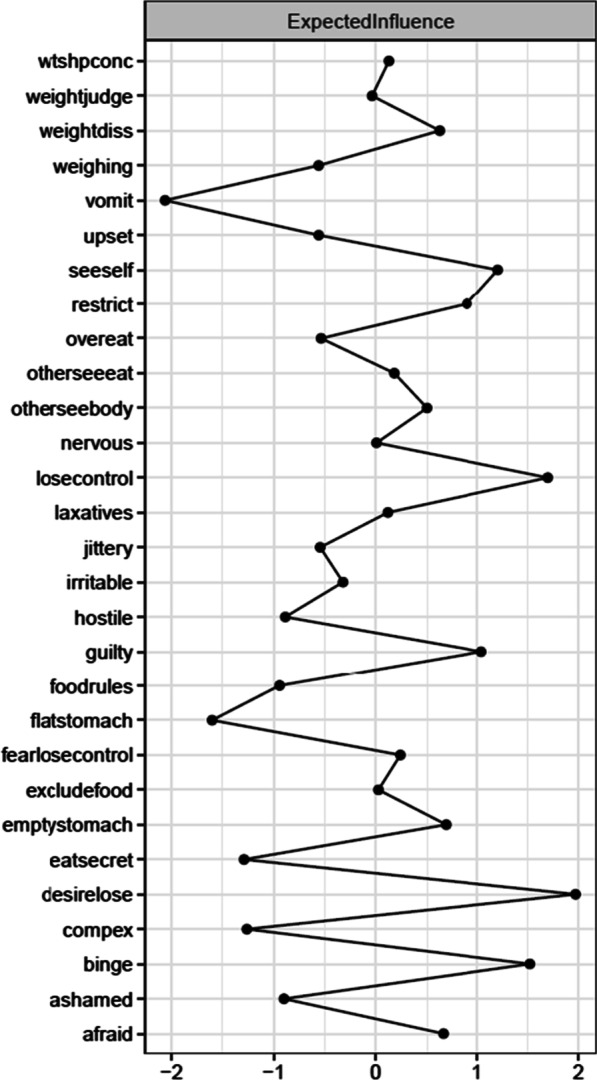
Network expected influence for the negative affect and eating disorder (ED) symptom network

**Fig. 3:**
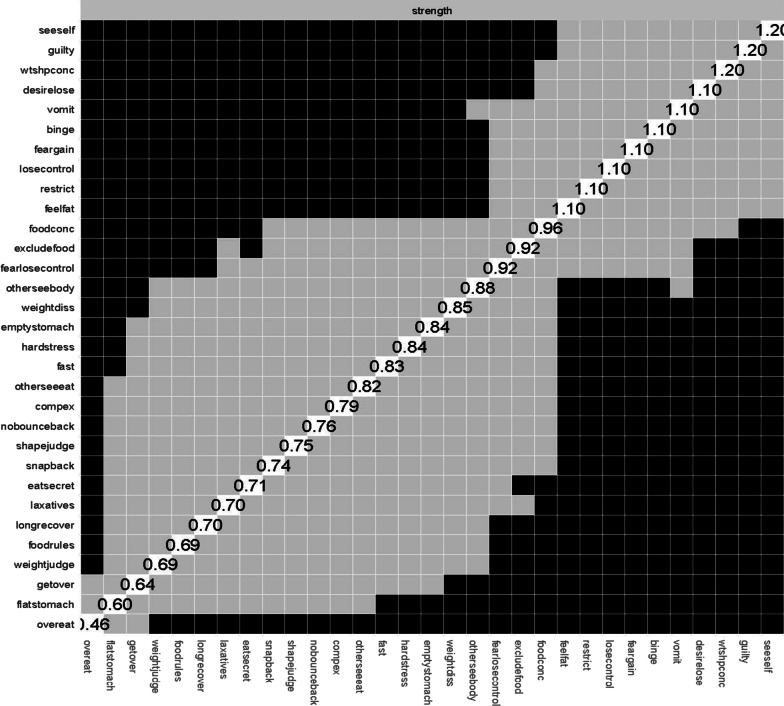
Node centrality difference test. Black squares indicate statistically significant differences between nodes at *p* < .05

Prior to data analysis, we examined raw data and did not find any outliers. Network stability was excellent (EI coefficient = 0.75; ES coefficient = 0.75). The item with the strongest EI was strong desire to lose weight (*desirelose,* ED symptom, EI = 1.97). This item had significantly higher EI than 82% of the other items in the network. The second item with the highest EI was definite fear of losing control overeating (*losecontrol,* ED symptoms, EI = 1.70). This item had significantly higher EI than 79% of the other items in the network. The third item with the highest EI was binge eating episodes (*binge,* ED symptom, EI = 1.52)*.* This item had significantly higher EI than 79% of the other items in the network. See Fig. 1 for the negative affect and ED symptom network, Fig. 2 for the EI influence plot, and Fig. 3 for the EI difference test plot.

### Bridge symptoms

**Fig. 4:**
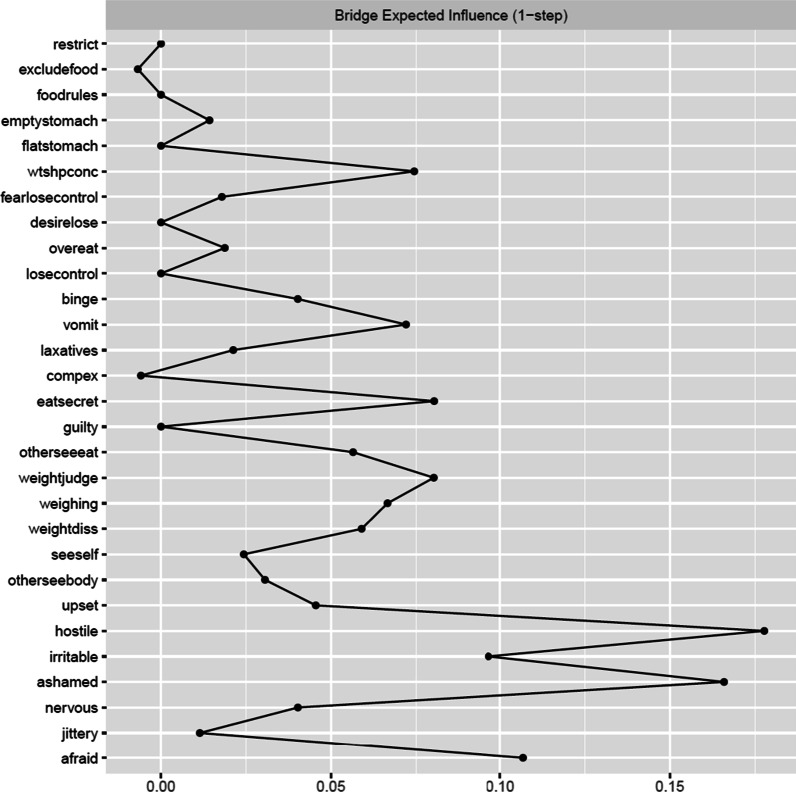
Bridge expected influence plot for negative affect and eating disorder (ED) symptoms

**Fig. 5:**
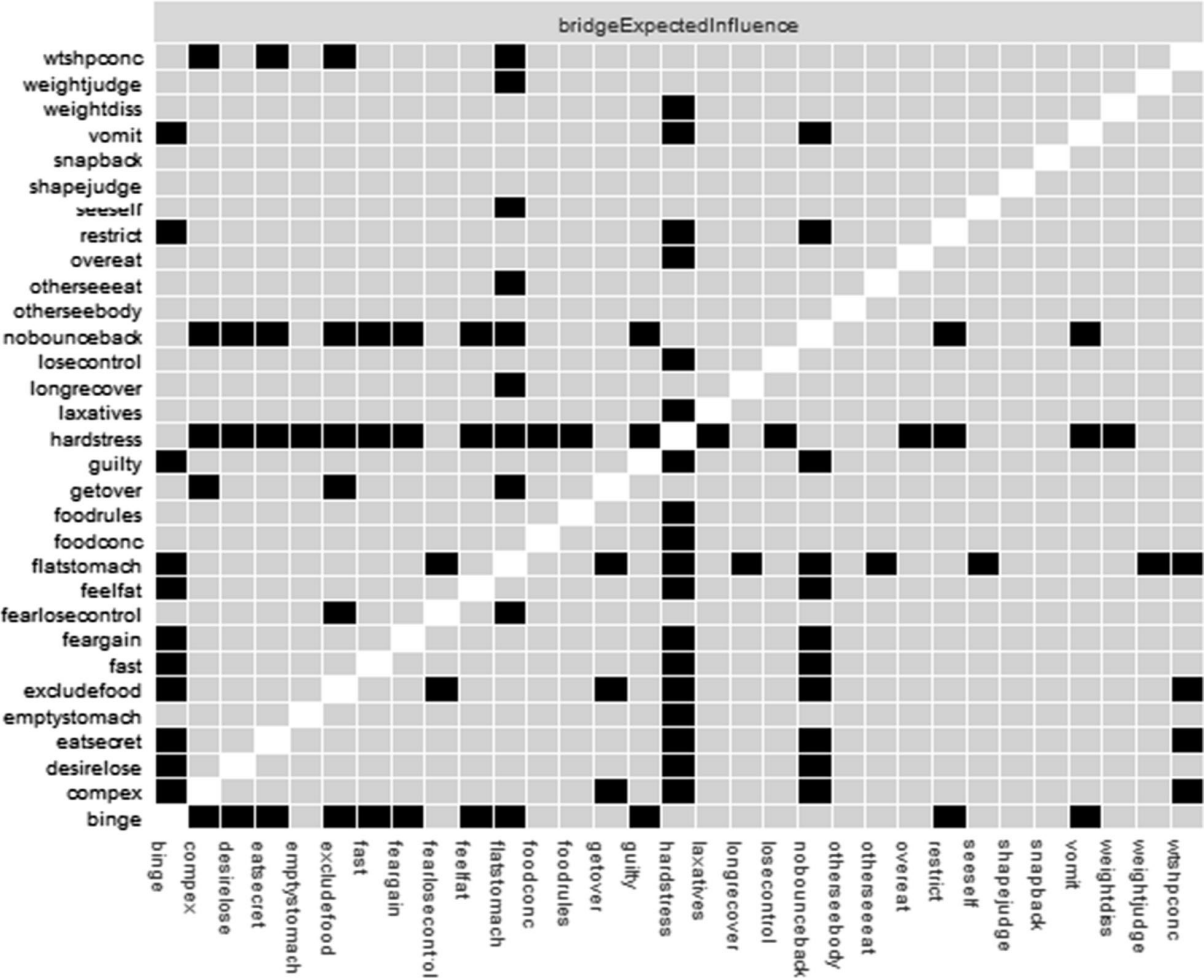
Bridge expected influence centrality difference tests

Bridge EI was acceptable (BEI coefficient = 0.44). The node with the highest bridge EI was the negative affect item, hostility (*hostile*, NA, bridge EI = 0.18). This item had significantly higher bridge EI than 65% of other items in the network. It was most strongly connected with the ED symptoms of vomiting (*vomit*, part *r* = 0.07) and eating in secret (*eatsecret*, part *r* = 0.07). The second item with the highest bridge EI was the negative affect item, shame (*ashamed*, NA, bridge EI = 0.17). This item had significantly higher bridge strength centrality than 65% of other items in the network. This item was most strongly positively connected with the ED symptoms of preoccupation with shape or weight making it difficult to concentrate (*wtshpconc,* part r = 0.07) and weight influencing self-judgment (*weightjudge*, part r = 0.02). See Fig. 4 for the bridge EI plot and Fig. 5 for the bridge expected influence difference test plot.

## Discussion

This is the first study to examine a network of negative affect and ED psychopathology among college students in Iran. We identified that hostility and shame were central bridge symptoms in a network of negative affect and ED psychopathology. We also found that the most central symptoms in the network were desire to lose weight, definite fear of losing control over eating, and binge eating episodes.

### Bridge symptoms in a network of negative affect and ED psychopathology

Hostility was a central bridge symptom, and bridged with vomiting and eating in secret. Notably, this finding is unique to our non-clinical Iranian college sample, as past research conducted among individuals with EDs in the US [51] did not find hostility to be a central bridge symptom. Hostility is considered to be an emotion that is commonly experienced among Iranian young adults [67]. Research suggests that Iranian individuals may experience hostility due to pressure because of low socioeconomic status, economic difficulties, and substance misuse [67]. The current study suggests that hostility links negative affective states and ED psychopathology in our sample. Individuals in Iran may find that hostility is often high [67], and may engage in ED behaviors such as eating in secret and vomiting to avoid and/or control high levels of hostility [25, 68, 69].

In line with the US network research [51], shame was another central bridge symptom, bridging with preoccupation with shape or weight and weight-related self-judgment. This may be because certain individuals may manipulate their body weight/shape in attempts to reduce shame (e.g., controlling their shape/weight/food to feel better about themselves). Notably, previous non-network research [28, 70–72] suggests that some individuals may engage in ED behaviors and manipulation of shape/weight to cope with shame. However, engaging in ED behaviors to cope with shame may eventually increase shame (e.g., regarding eating, shape and weight), setting up a vicious cycle [52]. Overall, our findings and those of others suggest that shame may have an important role in initiating ED psychopathology [26, 27, 29, 70, 73, 74].

### Central symptoms in a network of negative affect and ED psychopathology

We found that desire to lose weight was a central symptom in the ED psychopathology and negative affect network, which is consistent with previous research among US and Italian societies [33, 41, 43, 46–49]. This finding is consistent with another finding in Iranian college students where thin-ideal internalization was implicated in ED psychopathology [5]. An important implication of our finding is that Western sociocultural models [i.e., dual-pathway [13]; tripartite influence [75] in which thin-ideal internalization results in ED psychopathology may be relevant to non-Western societies such as Iran [5]. Further studies need to explore whether Western sociocultural models apply to non-Western populations such as Iran.

Consistent with network research in US samples [36, 49], definite fear of losing control over eating was another central symptom. Notably, this is the first study to date suggesting that fear of losing control over eating is a central symptom in an Iranian sample. Overall, this finding suggests that fear of losing control over eating is an important aspect of EDs in Iran in the context of negative affect. It can be noted that fear of losing control over eating is a relevant construct to all ED diagnoses [49, 76] and common among Iranian college students, with 31% of Iranian college students reporting loss of control eating [77].

Finally, binge eating was another central symptom. Previous research in US societies has had mixed results regarding whether behavioral symptoms of EDs, such as binge eating, are central, with some studies suggesting that binge eating is central to ED networks [41, 78], but others suggesting that binge eating is peripheral to ED networks [39, 45]. Our findings suggest that binge eating may be central in ED networks in Iran in the context of negative affect. Of note, the F-EDE-Q assesses objective binge eating (using only one item), but does not assess subjective binge eating. Therefore, it is not clear the extent to which the current findings apply to subjective binge eating. Future studies should be conducted using other scales which assess both subjective and objective binge eating with multiple items, such as the Farsi Eating Pathology Symptoms Inventory (F-EPSI; [79] and the Farsi Loss of Control Over Eating Scale (F-LOCES; [2]).

Interestingly, we did not find definite fear of losing control over eating and binge eating to be central symptoms in a previous study [4] in which we used the same data. This may be because in the previous study we examined a network of depression and ED symptoms. The centrality of symptoms in networks is largely dependent on the variables that are included in these networks. In the previously published paper, we used the Farsi Beck Depression Inventory-Second Edition (F-BDI-II; [80]). This inventory assesses four aspects of depression (cognitive symptoms, affective symptoms, motivational symptoms, and somatic symptoms). That is, the measures (i.e., F-BDI-II [80] vs. NA [60]) we used in two manuscripts included different symptoms (depression symptoms such as pessimism, past failure, crying, etc. vs. negative affective states such as hostility, shame, etc.).

### Clinical implications

Findings from the current study suggest that future interventions aimed at individuals high in negative affect and ED psychopathology in Iran should evaluate targeting hostility and shame, as network theory would suggest that targeting these states may weaken the association between negative affect and ED psychopathology. Self-compassion-based therapy may be a promising intervention to study in future research, as this type of therapy has been shown to reduce hostility, shame, and ED symptoms [81–83]. However, this type of intervention has not yet been evaluated in Iran. Future research should investigate adapting this type of intervention for use in Iran.

Network theory predicts that clinical interventions targeting central symptoms should result in reductions in other symptoms in the network [31, 33, 62, 84]. Although no research to date has tested whether targeting central ED symptoms improves ED interventions, research does suggest that central symptoms predict ED outcomes [33, 46, 84]. Findings thus suggest that future research should evaluate prevention programs that target desire to lose weight, fear of losing control over eating, and binge eating in Iran. The Body Project [85] is a prevention intervention that targets desire to lose weight and has been found to decrease risk for developing ED symptoms and negative affect among college samples [86, 87].

### Limitations and future directions

The study has several strengths. To our knowledge, this is the first network study examining symptoms that may bridge negative affect and ED symptoms in Iran. However, it is worth mentioning some limitations. First, several ED psychopathology symptoms are affective in nature (e.g., fear of weight gain, fear of losing control over eating, feeling guilty about eating), which may have influenced findings. Specifically, the extent to which findings relate to generic affective states vs. ED-related affective states is unclear. Second, similar to most network studies, this study was cross-sectional. Although other studies have shown that network structures among cross-sectional and longitudinal studies do not differ [39, 44, 56], to fully conceptualize how ED symptoms and negative affect symptoms dynamically relate to one another, longitudinal network analyses are needed [56]. Additionally, the results of the current study are limited by the measures used. For example, previous studies reported that risk factors such as muscular-athletic-ideal internalization also contribute to ED psychopathology among college men and women in Iran [5–7]. However, the EDE-Q did not assess muscularity/exercise-oriented attitudes and behaviors [88]. Therefore, future research should examine a network of ED symptoms using measures that include muscularity and exercise constructs (i.e., Farsi Eating Pathology Symptoms Inventory ([F-EPSI]; [79]). Relatedly, we did not include positive affect in the model. Lastly, another limitation is the use of a non-clinical sample with a narrow range of F-EDE-Q scores (i.e., 0.16–3.23). However, other studies have found that network structures (e.g., centrality of specific ED symptoms, edges) among non-clinical and clinical ED samples are similar [46, 47]. Furthermore, examining networks in non-clinical samples may have important implications for prevention research.

## Conclusion

Our findings suggest that negative affective states of hostility and shame may increase vulnerability to ED psychopathology in Iran. These findings are somewhat consistent with findings in US samples, where shame bridged negative affect and ED psychopathology. Unique to our sample, hostility also emerged as a central bridge symptom. Findings have important implications for ED prevention programs in Iran that should be examined in future research.

## Data Availability

The data that support the findings of this study are available on request from the first author.

## Abbreviations

ED: Eating disorder
US: United States
F-EDE-Q: Farsi-eating disorder examination-questionnaire
F-PANAS: Farsi-negative affect
F-EPSI: Farsi-eating pathology symptoms inventory
F-LOCES: Farsi-loss of control over eating scale
EI: Expected influence
ES: Edge stability
